# Retraction Note: Downregulation of the Rho GTPase pathway abrogates resistance to ionizing radiation in wild-type p53 glioblastoma by suppressing DNA repair mechanisms

**DOI:** 10.1038/s41419-025-08071-4

**Published:** 2025-09-23

**Authors:** Yuli Thamires Magalhaes, Viktor Kalbermatter Boell, Giovanna Duo Cardella, Fabio Luis Forti

**Affiliations:** Laboratory of Biomolecular Systems Signaling, Department of Biochemistry, Institute of Chemistry, University of São Paulo, São Paulo, Brazil

**Keywords:** Cytoskeleton, DNA damage and repair

Retraction Note to: *Cell Death and Disease* 10.1038/s41419-023-05812-1, published online 21 April 2023

The Editor-in-Chief has retracted this article. After publication, a number of concerns were raised, specifically:

- p53 bands in figure 1D is described differently in the original western blot image in the supplementary material
- seven panels were found to overlap within figure 2C, 2J and 2M
- a western blot was found to overlap between figure 7 and supplementary figure 2A
- the actin band in figure 7G is described differently in the original western blot image in the supplementary material
- Figures 5J and supplementary figure 3D appear to overlap
- Figure 6F sip53+ RhoA-GTP and RhoB-GTP appear to overlap with editing

A panel in figure 2C and two panels in figure 2J appear to overlap with a previously published protocol by the same authors [1].

The authors were unable to provide sufficient original data or a sufficient response to concerns. The Editor has lost confidence in the data and conclusions of this article.

Author Fabio Luis Forti disagrees with this retraction. Giovanna Duo Cardella could not be contacted by the publisher. Authors Yuli Thamires Magalhaes and Viktor Kalbermatter Boell did not respond to the publisher regarding this retraction.
